# Scaling Up Paediatric HIV Care with an Integrated, Family-Centred Approach: An Observational Case Study from Uganda

**DOI:** 10.1371/journal.pone.0069548

**Published:** 2013-08-06

**Authors:** Emmanuel Luyirika, Megan S. Towle, Joyce Achan, Justus Muhangi, Catherine Senyimba, Frank Lule, Lulu Muhe

**Affiliations:** 1 Mildmay Uganda, Kampala, Uganda; 2 Independent Health Consultant, Mumbai, India; 3 World Health Organization Regional Office for Africa, Brazzaville, Republic of Congo; 4 World Health Organization Headquarters, Geneva, Switzerland; Vanderbilt University, United States of America

## Abstract

Family-centred HIV care models have emerged as an approach to better target children and their caregivers for HIV testing and care, and further provide integrated health services for the family unit’s range of care needs. While there is significant international interest in family-centred approaches, there is a dearth of research on operational experiences in implementation and scale-up. Our retrospective case study examined best practices and enabling factors during scale-up of family-centred care in ten health facilities and ten community clinics supported by a non-governmental organization, Mildmay, in Central Uganda. Methods included key informant interviews with programme management and families, and a desk review of hospital management information systems (HMIS) uptake data. In the 84 months following the scale-up of the family-centred approach in HIV care, Mildmay experienced a 50-fold increase of family units registered in HIV care, a 40-fold increase of children enrolled in HIV care, and nearly universal coverage of paediatric cotrimoxazole prophylaxis. The Mildmay experience emphasizes the importance of streamlining care to maximize paediatric capture. This includes integrated service provision, incentivizing care-seeking as a family, creating child-friendly service environments, and minimizing missed paediatric testing opportunities by institutionalizing early infant diagnosis and provider-initiated testing and counselling. Task-shifting towards nurse-led clinics with community outreach support enabled rapid scale-up, as did an active management structure that allowed for real-time review and corrective action. The Mildmay experience suggests that family-centred approaches are operationally feasible, produce strong coverage outcomes, and can be well-managed during rapid scale-up.

## Background

Nearly 150,000 children are living with HIV in Uganda, and the majority of children are under five years of age [1]. Vertical transmission accounts for an estimated 18% of new infections nationally [2], and an estimated 53% of HIV-infected women receive antiretroviral to prevent mother-to-child transmission (PMTCT) [3]. While nearly 60% of HIV-exposed infants are tested within 12–16 months after birth, there is high loss to follow-up among exposed infants [1]. Yet despite the clear need to scale-up comprehensive prevention, care, and treatment for HIV-exposed and infected infants, paediatric services have lagged behind adult care both in Uganda and internationally [4]. Challenges to scale-up have included: poor linkages from PMTCT programmes and the subsequent missed opportunities for identification during postnatal and child health care; challenges of early infant diagnosis; the prioritization of adult treatment and subsequent lag in availability of paediatric antiretroviral dosages; and limited paediatric expertise amongst healthcare providers [4]–[12].

Family-centred approaches to HIV care have emerged as an effort to better target children and their caregivers for testing and care, and to provide integrated, comprehensive HIV and health services that support the range of a family unit’s care needs. A family-centred approach seeks to provide services within dynamic familial relationships, particularly given that infection often occurs in the familial context (e.g. sexual relations, pregnancy, breastfeeding). Family members are also often responsible for chronic care among HIV-infected individuals, and the impacts of HIV may affect the family beyond the HIV-infected individual, and are often intergenerational [13]–[14].

**Figure 1:**
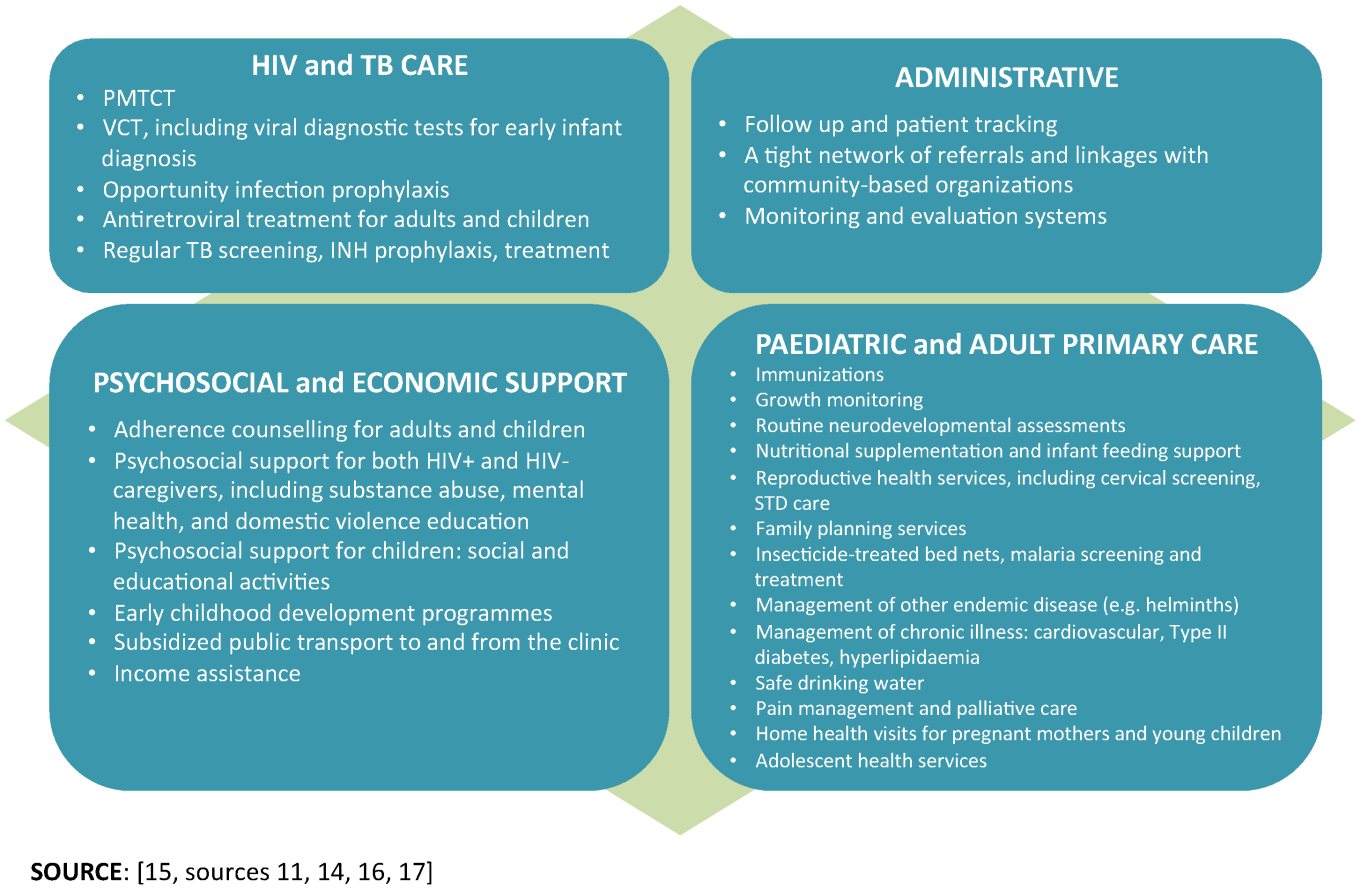
Possibilities for integrated service package for family-centred care. Figure displays recommended services listed within four sub-headings: HIV and TB care, paediatric and adult primary care, psychosocial and economic support, and administrative services.

In the past, paediatric HIV care, PMTCT, and ART have been funded and provided as stand-alone, parallel services [12]. Alternatively, family-centred care intends to integrate HIV services within the broader primary healthcare system and provide a comprehensive, ‘one stop’ service package for families, and increase access to paediatric services (Figure 1). Service beneficiaries move beyond the index clients to include spouse and/or sexual partners, children, family members, and community members in a facility’s catchment area [12]–[14], [17]–[18]. Family-centred HIV care should include timely maternal and paediatric HIV diagnosis, antiretroviral prophylaxis, co-trimoxazole prophylaxis, and long-term antiretroviral therapy for an entire family. It can also include a number of other health interventions, including treatment of opportunistic infections (OIs), infant feeding, sick and well child care, immunizations, malaria prevention and treatment, and tuberculosis interventions including isoniazid preventive therapy [12].

Thereby, a family-centred approach to HIV care is an optimal opportunity to capture paediatric clients at key entry points of care, including sexual and reproductive health services (e.g. prevention of mother-to-child transmission, PMTCT), postpartum care, paediatric and adult outpatient and inpatient departments (e.g. integrated management of child illness, IMCI), immunization clinics, and community-based health mobilization [15]–[17]. The challenge for family-centred approaches is to integrate HIV care into a wide continuum of services, particularly in an effort to capture children that are both symptomatic and asymptomatic, as well as those living with non-biological caregivers [15], [19]. In doing so, integrated HIV services are critical to progress in the Millennium Development Goals, and particularly the incidence of child morbidity and mortality (Goal 4), maternal mortality (Goal 5), and tuberculosis (Goal 6) [20].

Preliminary evidence on family-centred approaches demonstrates good outcomes in preventing mother-to-child transmission, increasing paediatric referrals, improving paediatric clinical outcomes, and supporting adherence and retention in HIV care [12], [15], [17]–[18]. Children, both HIV-infected and not, also indirectly benefit when their caregivers are treated, as evidenced by decreases in malarial and diarrhoeal episodes, hospitalizations, and mortality, as well as improvements in school enrolment and nutrition, and decreases in child labour [21]–[24]. This said family-centred HIV models vary widely in service delivery approach, beneficiary recruitment targets and methods, and level of service integration.

While there is interest in family-centred care models, there is a dearth of research sharing operational experiences. This paper seeks to examine good practices and enabling factors in a family-centred model scaled up in Uganda by a non-governmental organization, Mildmay Uganda, and identify key lessons and recommendations for a family-centred approach targeting paediatric care. By examining the operational model that contributed to this rapid scale-up of family-centred, integrated HIV care, this case aims to contribute to a wider discussion on increasing access to high-quality HIV prevention, treatment, and care services for children in resource-limited settings. Disseminating experiences in translating policy into practice aims to benefit policy makers, programme managers, implementation partners, and healthcare providers.

## Methods

### Case Study

In 2003 Mildmay Uganda began using a family-centred approach to integrate paediatric HIV care into adult HIV and maternal and child health services in its own centre, nine partner facilities, and ten community health facilities in six districts of Uganda’s Central region. These facilities included public and private (non-governmental and faith-based) institutions. The integrated package at Mildmay included the following services: (a) HIV diagnosis, prevention, prophylaxis, and treatment services; (b) reproductive and maternal health services, including PMTCT, family planning, and cervical cancer screening; (c) paediatric outpatient and inpatient services, including rehabilitation for severe acute malnutrition; (d) adolescent and adult outpatient and inpatient services; (e) commodity distribution for families enrolled in care (e.g. insecticide treated nets, safe water supplies); (f) specialist services including paediatric dental, adult and paediatric ophthalmology, occupational therapy, physiotherapy, and mental health; (g) TB screening and treatment; and (h) home-based follow-up and counselling support from community-based volunteers and community nurses.

### Documentation

Documentation efforts examined retrospectively how Mildmay and its partners utilized the family-based care approach to integrate HIV services in facilities. First, key informant interviews with programme managers, care providers, and patient families examined operational successes and challenges across sites. Documentation particularly examined Mildmay problem-solving around challenges noted in family-centred care literature and were analyzed focusing on the changes in key services approaches. Key informant interviews with patients are routinely conducted, with consent, within patient satisfaction and programme review processes. Given that it is routine and consent is obtained for use in programme research and evaluation there are no IRB or Uganda National Council for Science and Technology (UNCST) approvals involved.

Second, a key documents review examined programmatic strategy pieces, national paediatric HIV care policies, technical guidelines, and relevant memos, e.g. previous internal documentation work on cotrimoxazole scale-up and disclosure to children at Mildmay. Third, a desk study examined electronic Health Management Information System data maintained at the Mildmay main site between 1999 and 2010. Data collection tools were developed by experienced technical staff, and the same data set is used for reporting to the Ministry of Health, Centers for Disease Control and Prevention, Monitoring and Evaluation of the Emergency Plan Progress, monthly district reports, and other stakeholders. Key data points available at the time of intervention (2003) and at period of analysis (2010) were entered into Excel and analyzed using simple frequency distribution. These included the number of children enrolled in care and the number of children on cotrimoxazole prophylaxis and/or ART. All Mildmay Uganda clients provide informed consent for use of stored medical record data for future programme research and evaluation. As we only analysed aggregate, anonymous hospital administrative data, and the research only documented best practice and lessons learnt using routine data of consenting clients, no further ethics review was pursued.

### Limitations

As a retrospective and largely observational study, controls were not available to study the specific causality of family-centred care interventions on decreases in paediatric mortality and disease incidence.

## Results

**Figure 2:**
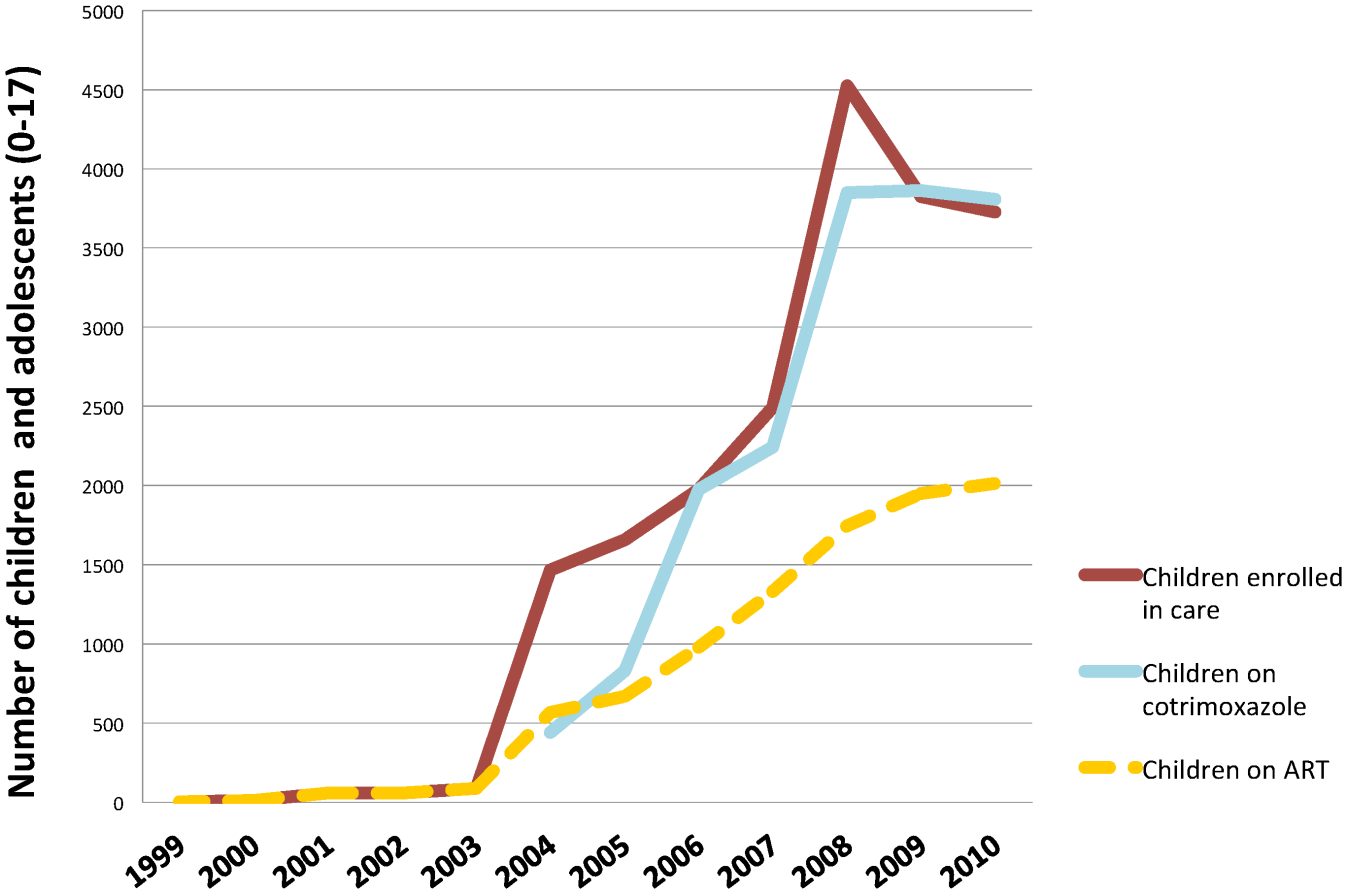
Cumulative trend of uptake of paediatric care after introduction of family-based approach at Mildmay main site in 2003. Figure demonstrates uptake trends from 1999 to 2010 for three key indicators: number of children and adolescents enrolled in HIV care, number on cotrimoxazole prophylaxis, and number on ART. Uptake data shows sharp increases for all three indicators at 2003, when the family-based approached was introduced at Mildmay.

Results demonstrate a significant increase in demand for, and utilization of, paediatric HIV care since the introduction of the family-centred care strategy. After the 2003 introduction of the family-centred approach at the Mildmay main site, the numbers of children enrolled in HIV care, receiving cotrimoxazole prophylaxis, and on antiretroviral treatment increased (Figure 2). The approach resulted in a 50-fold increase of families registered in HIV care at Mildmay and supported facilities (n = 70 to 3,653). Family units enrolled in care average two adults and two children. From 2003 to 2010, Mildmay experienced a 43-fold increase of children actively enrolled in care (n = 86 to 3726) and a 23-fold increase of children on ART (n = 86 to 2015). Mildmay was one of the first organizations to begin providing antiretroviral treatment for children in Uganda; between 1999 and 2003 a small number of children were enrolled in HIV care and receiving antiretroviral treatment. About half of these children at any given time during the review period were eligible to receive ART given the initiation criteria; of note, ART guidelines have since changed and all infected children under five are eligible to initiate ART. Additionally, the facility has achieved nearly universal paediatric cotrimoxazole coverage after prophylaxis scale-up began in 2004.

## Discussion

Service uptake results suggest that the family-centred approach captured numbers of HIV-infected children for enrolment in integrated care. At the time of this evaluation, nineteen percent of Mildmay’s clients (n = 22,000) were children, which is one of the highest proportions among Ugandan health facilities and exceeds the national target of 10% paediatric patients. It is certainly worth beginning a discussion by noting that the availability of free ARVs in Uganda in 2004 brought national momentum to treating both adults and children. However, we argue that Mildmay’s approach in encouraging families to access services together played a very important role in increasing numbers of children in care. The Mildmay paediatric caseload continues to exceed the national average per site; districts where Mildmay is implementing health systems strengthening support do not otherwise have an emphasis on family-centred care, and still have very low paediatric care coverage. Many sites in these districts with ART availability–and without the integrated family approach–still do not offer paediatric HIV care.

**Figure 3:**
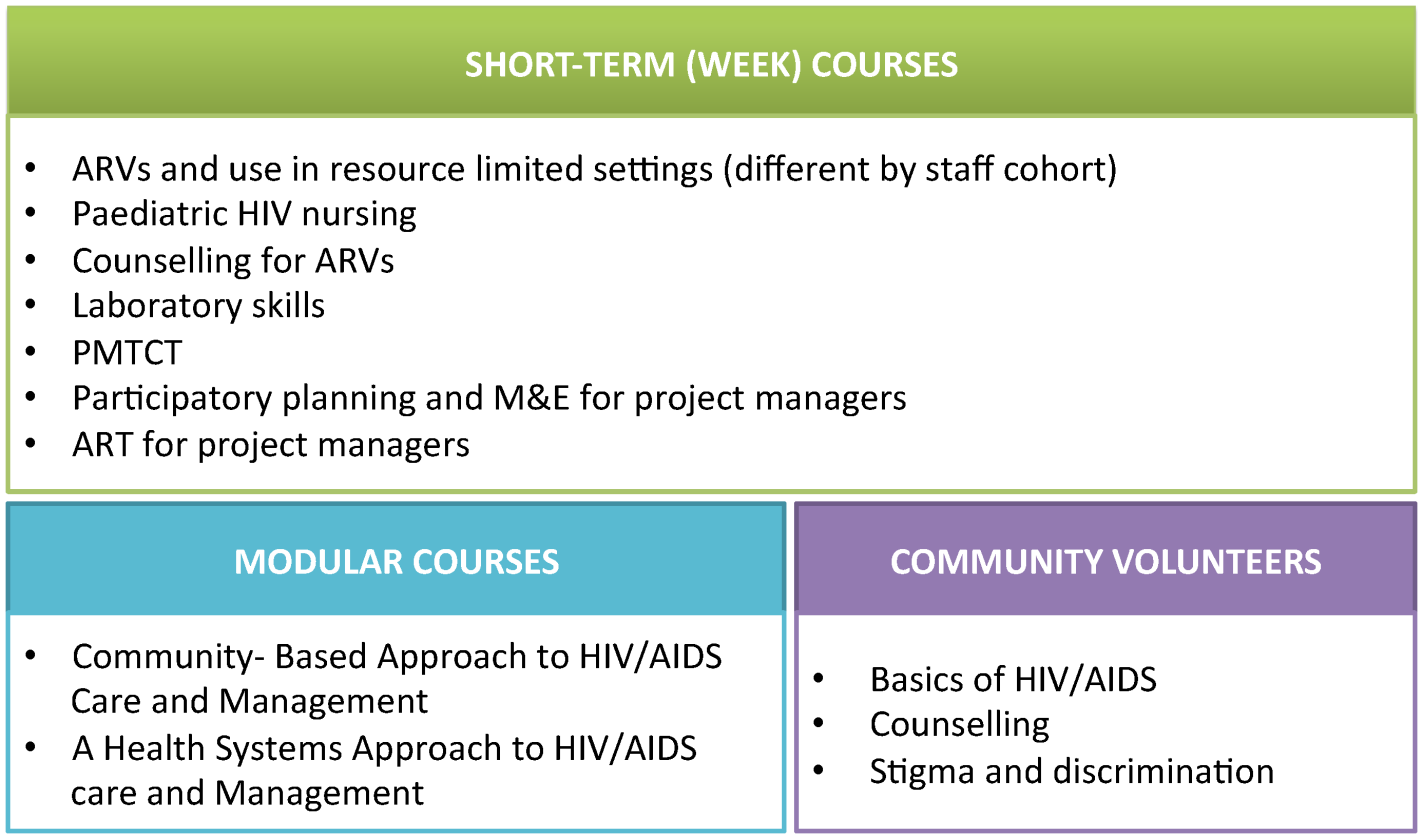
Capacity building for integrating family-centred care at partner facilities. Figure describes the courses designed by Mildmay and made available during partner facility service integration, as needs assessments determine. These include short-term (week) courses on skills sets like pediatric HIV nursing or laboratory skills, modular courses on more advanced subjects like community-based HIV care and health systems approaches, and training for community volunteers on HIV/AIDS basics and counseling skills.

As no facilities were offering paediatric HIV care at the time of scale-up, several measures were required to build capacity to provide paediatric care, to deliver comprehensive services, and to operate within the new integrated delivery approach. In supporting scale-up of family-centred care, Mildmay Uganda worked with identified partner facilities to: conduct needs assessments, provide capacity building for the multidisciplinary teams involved with service provision (Figure 3), advise facility restructuring as required for efficient patient capture and integrated service delivery, target the family networks of HIV-infected clients for enrolment into integrated health services, drive monitoring and evaluation, revise programmatic guidelines, and prepare supportive exit strategies with partner facilities.

### Operational Best Practices

Our qualitative examination of Mildmay’s approach highlights a number of best practices and enabling factors that have streamlined integrated care services provision at the facility, supported access at the community level, and provided for active management of the family-centred approach during rapid scale-up.

#### Incentivizing care seeking as a family

**Figure 4:**
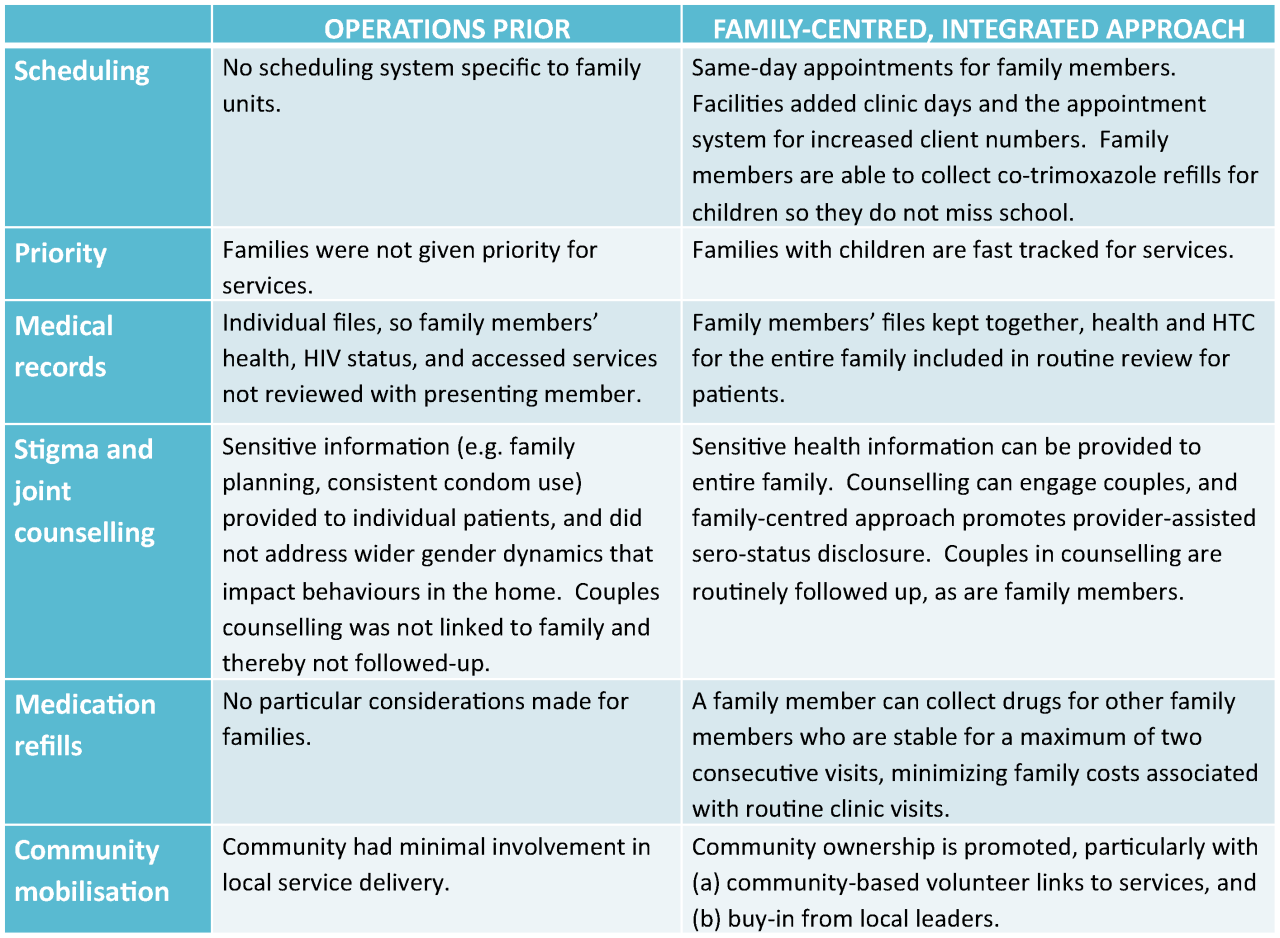
Service approach at health facilities before and after family-centred approach. Figure describes several service delivery components (e.g. scheduling, counseling, medication refills, and community engagement) before and after the family-centred approach. The figure demonstrates considerable effort to re-align the service approach to make it family-friendly, for example, same-day scheduling for families or fast-tracking families with children for services.

Facilities have reorganized their delivery approach to provide ‘one stop’ package of services that incentivizes care-seeking as a family (Figure 4). Within the family-centred approach, beneficiaries frequently cited client satisfaction during interviews as a key reason for seeking services. Patients can be referred within the same facility for different services, or re-scheduled as a family if additional services will require prolonged waiting time or the clinical services required are offered on another day of the week. Beneficiaries are given same-day appointments if the family chooses. Family members are also fast tracked for treatment if they come with children, an incentive for parents to initiate care with their children. Mildmay facilities also permit alternate drug collection by approved family members, so that families can acquire treatment when the index client is too weak or unable to go to the facility, or the child is in school. Facilities refer when necessary, and also link families to social support services including livelihood projects and education support for children. Integrated HIV services should also reduce household costs for seeking services at multiple facilities, or multiple visits for different service components; this data was not collected from clients but could be the focus of future research.

#### Provider-facilitated disclosure

Mildmay experienced a 30% increase in partner testing, and the disclosure rates increased from 30% to 70% in 72 months, following provider-assisted partner disclosure as an entry point into HIV care. The family-centred approach promotes provider-facilitated serostatus disclosure to significant others when the client is prepared, thus further enhancing the family support system. Counsellors provide a number of disclosure methods; clients can disclose in the presence of a counsellor, or can request the counsellor disclose to their parents in their presence, or others request time to disclose on their own. While index clients are encouraged to disclose to their family members, contact with families only occurs when the index client is ready. Additionally, 152 unique individuals in discordant relationships have been identified and supported. Follow-up with clients as family units has also enhanced continued counselling for couples.

#### Provider-initiated testing and counselling and early infant diagnosis integrated into triage

**Figure 5:**
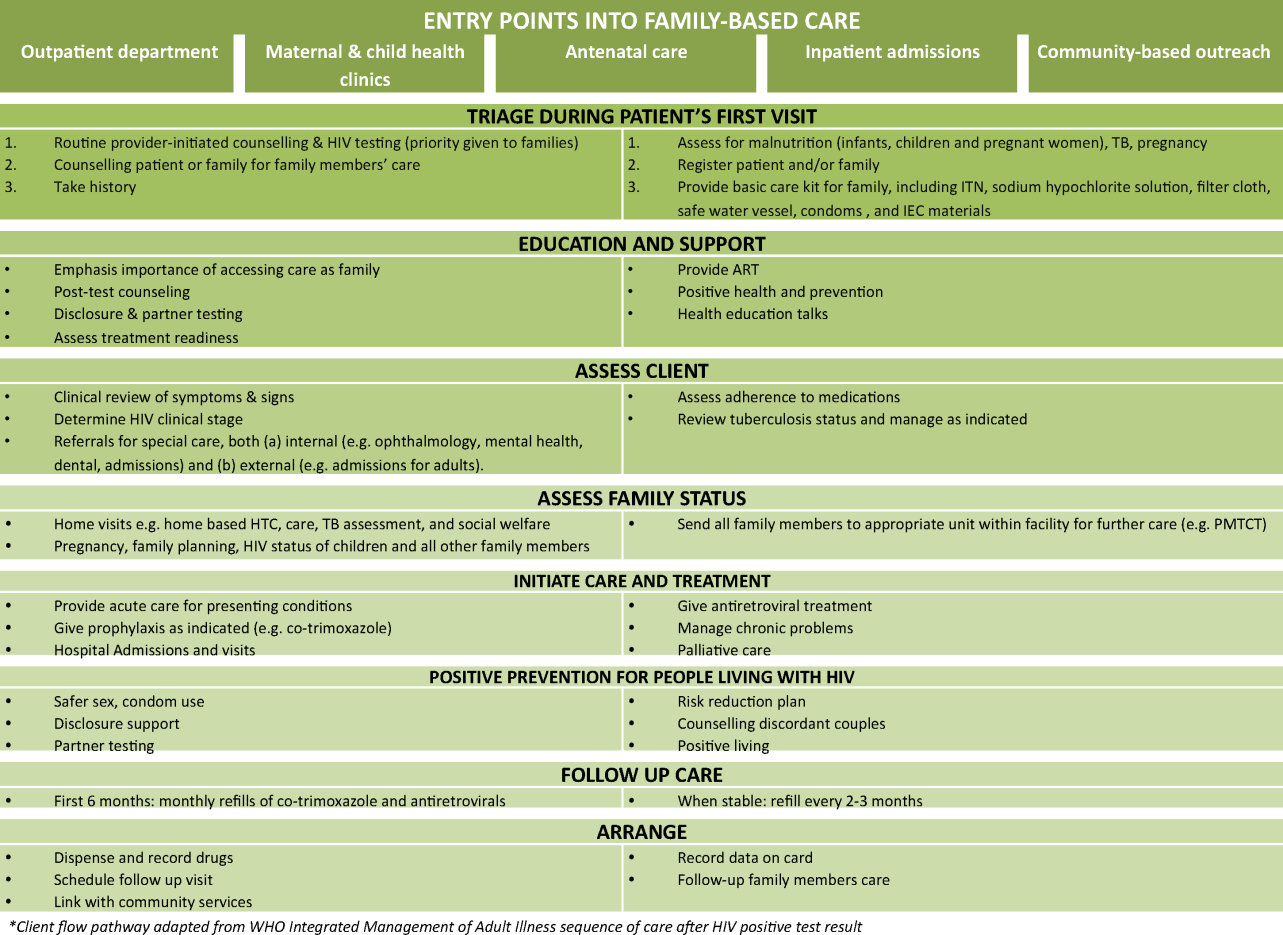
Client flow pathway into HIV care and treatment within family-centred approach. Figure demonstrates the patient flow at integrated facilities; the pathway has been adapted from the WHO Integrated Management of Adult Illness sequence of care. Entry points into family-based points including outpatient care, maternal and child health clinics, antenatal care, inpatient admissions, and community-based outreach. Clients then proceed through triage assessments, education and support as required, assessment of client and family health status, care and treatment as required, positive prevention for HIV-infected clients, and follow-up care services.

Routine provider-initiated testing and counselling (PITC) has been integrated into triage at key entry points to care, including antenatal care, outpatient clinics, and inpatient facilities. All staff members are trained on the client flow pathway for identified HIV-exposed and infected children (Figure 5). In post-test counselling, there is a strong focus on preparing the index client to bring other family members in for testing, counselling, and care. As women more women seek services in Mildmay facilities–particularly as primary caregivers coming for their child’s care–emphasis is placed on partners testing. Counselling urges family members to take drugs together at the prescribed time, and to encourage each other to observe care provider instructions and agreed plans with providers (e.g. safer sex, nutrition, treatment adherence). Patients and communities qualitatively attribute good adherence to supportive home environments.

Routine PITC is particularly strong with decentralized capacity for early infant diagnosis (EID). All supported facilities have been trained and equipped to collect dry blood spots, especially within postnatal care services. Dry blood spots are collected every two weeks from partner facilities, and sent centrally from Mildmay to Kampala-based laboratories. Turnaround for laboratory results is a maximum of 14 days. Staff members report that the timely return of test results fosters client trust in the availability of services, encouraging caregivers to bring children back for further management.

In addition to PITC strategies, free voluntary HIV testing and counselling (HTC) services are provided regularly at each facility. At every HTC encounter, the index client is encouraged to have their partner(s) and children tested as well.

#### Multi-pronged strategy for capturing paediatric referrals

Particular care is taken for parent-child and sibling referrals. At enrolment into care, information is captured about the index client’s children and if they have been tested. At every clinic visit, follow-up information regarding the children’s status is collected as part of the client review form. Similarly, for any child seen at Mildmay, reviews inquire about the siblings’ status and if they are in care. Parents and guardians are encouraged and supported to disclose HIV serostatus to older children. This has enhanced adherence and retention amongst this age group [25].

There are additional strategies to capture HIV-infected children living with non-parent or non-biological caregivers. Orphanages bring children in their care for testing, as do some schools after obtaining parental consent. Churches also liaise with Mildmay to conduct HTC for their congregations. Community-based volunteers target grandparent-run households to follow-up with children in care. Lastly, Mildmay’s waiting rooms host morning ‘health talks’ in the facility waiting area to capture clients.

#### Child-friendly service environment

Mildmay Uganda has created a child-friendly environment at its main health centre, including furniture, toys, play equipment, and on-duty child counsellors. Children’s play clubs organized at health facilities bring children together on specific days to interact with other children and receive support. The care at these play clubs varies by age group, and children are encouraged to give feedback on their experiences with treatment. Parents reported that the child-friendly environment is encouraging for the families, and they believe children are well-cared for centre.

#### Multi-disciplinary management team

A multi-disciplinary team of clinicians, nurses, social workers, counsellors, pharmacists, monitoring and evaluation team members, and psychosocial programming staff meet weekly to review operations and strengthen linkages across departments involved in delivering family-centred care. Weekly reviews also determine follow-up with clients who missed appointments, and also examine mortality data. The multi-disciplinary management designed has facilitated a number of innovations, including a particularly interesting mortality tracking system. In an effort to monitor client outcomes, while also recognizing the sensitivities around accurate mortality data, the Mildmay management team has instituted a memorial system to ensure mortality reporting. Throughout the year, monitoring teams compile the families who have lost a family member, and a memorial service is held each year for those who have passed away. Mildmay reports that the value attached to the memorial service has encouraged families to report deaths.

#### Task shifting in HIV care and community outreach support

Task shifting towards a nurse-led approach with community volunteer support has facilitated quality services, especially as client loads increase, by addressing gaps in skilled human resources, decentralizing services, facilitating service integration, and monitoring uptake in the community. Nurse-led community clinics can provide follow-up care to infants and children that do not require physician review, and for non-complicated cases can reduce wait time at facilities and minimize direct staff costs under management by nurse time instead of medical officer time. The proportion of child reviews by the nurse-led clinic has risen from 5% to 12% between 2007 and 2010; however wait times were not measured.

Mildmay’s family-centred care has trained community-based volunteers (CBV) and volunteer nurses to provide outreach and home-based care to a client load of ten patients, including home-based care, drug adherence support, follow-up visits for pregnant women and children, and mobilization for HTC. CBVs are often members of the Village Health Teams created by the Ministry of Health, some are local leaders, and many are expert patients. CBVs are managed by a local head, who reports directly to the health facility. CBV incentives include transport costs for scheduled activities, limited days of work in an effort to not overburden, and bicycles for CBVs who have served at least six months and regularly follow-up with at least ten patients.

Mildmay has also created mechanisms to promote community ownership. Community decision-making has been instituted at some facilities, and community members serve on the facility management committees that plan service integration. Quarterly meetings between community members and service providers review operations of the CBVs and health providers, clients’ drug taking and readiness to start treatment, and general services uptake. Additionally, local councils (LCs) provide political will and key linkages to services. LCs vet children for services and formally refer children to play clubs at the facility. Local authorities help supervise CBV operations in their villages.

### Considerations for Scale-up

These operational outcomes suggest that family-centred care is a feasible and effective method for capturing paediatric clients and providing comprehensive HIV care as a ‘one stop’ model for the family. While our case study highlights a number of best practices and enabling factors in the Mildmay experience, there are also noteworthy challenges and considerations for scaling up integrated care.

First, a number of resource investments are required for scale-up of integrated, family-centred care. The model relies on a secure procurement and supply chain system, and human resources for care and follow-up support. The most critical human resource requirement for scale-up was the creation of the multi-disciplinary management team, but otherwise staff was not added. In settings with limited staff numbers, clinics were encouraged to use task shifting and volunteers. As this paper identifies, one significant challenge to integrated care has been traditional funding of vertical streams, e.g. paediatric HIV, maternal care, and adult HTC. Resource requirements and costing have not been systematically studied in this case study, but are an important area for continued research.

Second, staffing levels and facility infrastructure must be prepared to provide quality services for increased patient loads, balance workloads for service providers, and minimize client waiting time. Reorganization of space and patient flows may be required to provide adequate triage, counselling, and waiting space. As discussed, Mildmay utilized task-shifting for additional human resources support, and also adopted additional clinical days and a new scheduling system to better manage additional clients.

Third, family-centred care requires staff training and support for paediatric and family-oriented care, and attrition after orientation to the approach hinders programme scale-up. As discussed, Mildmay instituted a multi-disciplinary management team to ensure more consistent communications and support across facilities and provider units. The management team also had to continue to identify emerging training needs (Figure 3) for health workers, volunteers, and managers after the initial integration period.

Fourth, the approach required planned logistical support for community-based activities like outreach and mobilization (e.g. transport for community based volunteers, which was not originally planned for). This is particularly challenging with outreach for families requiring additional support due to extreme poverty or difficult accessibility, and when children are living among multiple households that require several points of contact with caregivers. These situations may necessitate outreach clinics and care provision. Finally, continued stigma and discrimination around HIV and care seeking persists in some communities–for example, reluctance to ‘waste time’ on the care of HIV-infected orphans–and requires ongoing mobilization activities and local advocacy.

The growing programmatic and policy interest around family-centered care models emphasizes the need to share scale-up results and challenges, as this paper aims to do. As an emerging model of practice, there is considerable scope for further research in effective delivery, including leadership and capacity building in facilities that re-align service delivery towards the family-centred model, and the impact of integrated care on paediatric clinical outcomes.

### Conclusions

The family-centred approach at Mildmay has integrated paediatric early diagnosis, prevention, treatment, and care into outpatient and inpatient care, maternal and child health services, and HIV care. Operationally, a number of best practices have facilitated high-quality services and decentralized access during rapid-scale up in partner facilities. The Mildmay experience highlights the critical need to re-align care provision towards integrated service packages that incentivize care-seeking as a family, and in child-friendly environments. Mildmay’s approach was aided by provider-initiated or facilitated testing and counselling to target index clients and families for care, and task-shifting towards nurse-led clinics with community outreach support. These community outreach mechanisms diversified service entry and provided critical adherence support, including targeted volunteer efforts at the household level; engagement with local leaders; and service outreach to churches, schools, and orphanages. Multi-disciplinary team management and tracking mortalities through an annual memorial service further enabled further facilitated informed scale-up. The Mildmay scale-up experience emphasizes that family-centred care approaches can be operationally feasible, and it is a promising approach for producing positive programmatic and uptake outcomes for paediatric HIV/AIDS care.
